# Impact on survival of glioblastoma patients in relation to the imaging of the peri-surgical area: a multi-parametric diffusion MRI, perfusion MRI, and [^11^C]MET PET study

**DOI:** 10.1093/bjr/tqag117

**Published:** 2026-05-20

**Authors:** Chris W J van der Weijden, Hanne-Rinck Jeltema, Michiel Wagemakers, Roelien H Enting, Gilles Stormezand, Miranda C A Kramer, Bart R J van Dijken, Anouk van der Hoorn

**Affiliations:** Department of Radiology, Medical Imaging Center, University Medical Center Groningen, University of Groningen, Groningen 9713 GZ, The Netherlands; Department of Nuclear Medicine and Molecular Imaging, Medical Imaging Center, Groningen 9713 GZ, The Netherlands; Department of Neurosurgery, University of Groningen, University Medical Center Groningen, Groningen 9713GZ, The Netherlands; Department of Neurosurgery, University of Groningen, University Medical Center Groningen, Groningen 9713GZ, The Netherlands; Department of Neurology, University Medical Center Groningen, University of Groningen, Groningen 9713GZ, The Netherlands; Department of Nuclear Medicine and Molecular Imaging, Medical Imaging Center, Groningen 9713 GZ, The Netherlands; Department of Radiation Oncology, University Medical Center Groningen, University of Groningen, Groningen 9713GZ, The Netherlands; Department of Radiology, Medical Imaging Center, University Medical Center Groningen, University of Groningen, Groningen 9713 GZ, The Netherlands; Department of Radiology, Medical Imaging Center, University Medical Center Groningen, University of Groningen, Groningen 9713 GZ, The Netherlands

**Keywords:** glioblastoma, perfusion MRI, diffusion MRI, intravoxel incoherent motion, [^11^C]methionine PET, overall survival, biomarker

## Abstract

**Objectives:**

Glioblastoma (GBM) is an aggressive brain tumor with poor prognosis; recurrence near the surgical cavity is common despite multimodal-treatment. Conventional MRI lacks accuracy for early detection of recurrence and cannot reliably differentiate progression from pseudoprogression. This prospective-study evaluated whether <72 h post-surgery imaging biomarkers from diffusion-weighted imaging (DWI), intravoxel incoherent motion (IVIM), dynamic-susceptibility contrast (DSC) MRI, and [^11^C]Methionine PET (MET PET), could predict progression-free survival (PFS) and overall survival (OS).

**Methods:**

A prospective cohort of 21 GBM patients underwent multimodal imaging. Metrics from 0 to 5 and 5 to 10 mm peri-surgical rims and contralateral white matter were analyzed with Spearman correlations and Cox regression.

**Results:**

IVIM diffusion coefficient D in the 0-5 mm rim correlated positively with OS (*r = *0.821, *P =* .023), whereas pseudo-diffusion D* in the 5-10 mm rim correlated negatively (*r* = −0.786, *P =* .036), consistent with longer survival with lower microvascular perfusion/higher lower tissue density. Cox regression showed a significant association between SUVmax MET PET uptake and OS (HR = 13.022, *P =* .044), indicating a lower SUVmax MET PET corresponds with a longer OS.

**Conclusions:**

IVIM-derived metrics and MET PET SUVmax may serve as prognostic biomarkers of OS after GBM surgery. Despite small sample size and heterogeneous acquisition, the associations warrant validation in larger, standardized cohorts and exploration for personalized post-operative risk stratification.

**Advances in knowledge:**

This study shows that IVIM-captured microvascular characteristics and MET PET SUVmax <72 h post-surgery predict OS, outperforming conventional MRI and DSC metrics. It suggests that both vascularity near the cavity and residual-tumor signal may drive recurrence risk more than residual-tumor signal.

## Introduction

Glioblastoma’s (GBMs) are infiltrating brain tumors with a median life expectancy of 15 months.^1^^,^^2^ Treatment consists of maximal safe surgical resection, followed by concomitant chemoradiotherapy and adjuvant chemotherapy. Despite treatment, tumor recurrence inevitably occurs, contributing to the dismal prognosis of GBM. In the majority of cases, tumor recurrence occurs adjacent to the resection cavity, within the radiotherapy target volume. It is hypothesized that this is due to tumor cells infiltrating beyond imaging and neurosurgical margins which ultimately become resistant to chemoradiotherapy, leading to treatment failure. Timely recognition of tumor recurrence therefore is essential to allow future research or stop an ineffective treatment with its side-effects.

Current treatment response assessment with conventional MRI, timed at 3- to 6-month intervals, has several limitations and falls short on early identification of treatment failure.^3^ Conventional MRI, consisting of pre- and post-contrast T_1_-weighted and T_2_-weighted sequences, is unable to demonstrate the infiltrative margin of GBMs. Furthermore, conventional MRI can only detect tumor recurrence at late stages once new contrast enhancing lesions have reached. Lastly, early recognition of tumor recurrence is further hindered by treatment effects mimicking progressive disease (pseudoprogression), which are known to occur in one-third of patients.^4^ Earlier prediction of progression would allow a more personalized treatment plan in the future and enhance disease prognosis.

More advanced imaging techniques such as perfusion MRI, diffusion MRI, and amino PET are promising to overcome the limitations of conventional MRI.^3^^,^^5–7^ As tumors consume more energy than other tissue, parameters like oxygen extraction fraction (OEF) or the cerebral metabolic rate of oxygen (CMRO_2_),^8^ extracted from perfusion MRI might enable a better prediction of tumor-progression. Furthermore, indices for cellular density can be extracted from diffusion MRI.^9^^,^^10^ As tumors have a high cellular density, higher than healthy brain, this could be an important parameter. Using intra-voxel incoherent motion (IVIM), perfusion derived parameters might even be extracted from diffusion MRI, by exploiting the perfusion effects on diffusion images caused by the use of low *b*-values.^11^ Amino acid PET tracers, including [^11^C]methionine (MET), are transported through L-amino acid transporters (LAT), which are upregulated in tumors.^12^ The transport of MET into the cells is driven by concentration gradient of low intracellular methionine compared to high extracellular methionine. The low intracellular methionine concentration is due to intracellular metabolism of MET, which therefore reflects proliferation activity.

While OEF, CMRO_2_, IVIM, and MET PET are highly promising for differentiating tumor progression from pseudoprogression, the potency of these proxies for predicting tumor progression is not yet known. Therefore, this study aims to determine the peri-surgical parameters derived from DWI, IVIM, dynamic susceptibility contrast (DSC), and [^11^C]MET PET on post-surgery (<72 h) scans for progression-free survival time and overall survival time.

## Methods

### Subjects

Patients included in this prospective study were >18 years of age with a new primary GBM, scheduled to undergo standard treatment consisting of surgical resection followed by concomitant chemoradiation and adjuvant temozolomide chemotherapy according to the Stupp et al.’s^2^ protocol. Patients were excluded if they had a recurrent or secondary glioblastoma, other intracranial tumor, infratentorial GBM, prior brain surgery, or prior irradiation of the head. Written informed consent was obtained from all study participants. The study was approved by the Medical Ethics Review Committee of the University Medical Center Groningen in accordance to the Helsinki Declaration (METc number METc2017/513). Progression was based on comprehensive radiological assessment of the full MRI examination, including unequivocal tumor growth and/or new or increasing contrast enhancement, with clinical context and possible treatment-related effects considered where relevant. The progression-free survival (PFS) was defined as the time between tumor resection and scan with first tumor progression and OS time was the time between tumor resection and death.

### Image acquisition

All MRI scans were acquired on a 3T Siemens Prisma MR scanner equipped with a 64-channel head coil. The scan protocol consisted of structural MRI T_1_w MPRAGE (TR = 2300 ms; TE = 2.32 ms; TI = 900 ms; flip angle = 8°; voxel size = 0.9 × 0.9 × 0.9 mm; TA = 5:21), T_2_w-FLAIR SPACE (TR = 5000 ms; TE = 391 ms; TI = 1800 ms; flip angle = 90°; voxel size = 1.0 × 1.0 × 1.0 mm), and T_1_w post contrast (parameters identical to those of pre-contrast T_1_w), diffusion weighted imaging (DWI EPI; TR = 4500 ms; TE1 = 63 ms; TE2 = 106 ms; 3 orthogonal diffusion directions; 10 *b*-values = 0, 20, 40, 60, 80, 100, 250, 500, 750, 1000 s/mm^2^; voxel size = 1.1 × 1.1 × 4.0 mm; TA = 10.18), and perfusion MRI (PWI) using dynamic susceptibility contrast (DSC EPI; TR = 1780 ms; TE = 30 ms; flip angle = 90°; voxel size = 0.9 × 0.9 × 4.0 mm; TA = 1:57) using a bolus injection of 0.2 mL/kg gadolinium with a speed of 4 mL/s and flushing of 40 mL of 0.9% NaCl at a velocity of 4 mL/s.

[^11^C]Methionine PET was acquired on a Siemens Biograph mCT PET-CT scanner, starting with a low-dose CT for attenuation correction, followed by the MET PET scan. Individual doses of [^11^C]Methionine were prepared on site according to GMP quality assurance criteria. A dose of 200 MBq [^11^C]Methionine was injected 20 min prior the start of a 5-min static PET scan.^13^ PET images were corrected for randoms, dead-time, scatter, decay, and attenuation and reconstructed using TrueX + TOF with 3 iterations and 21 subsets in 1 frame (1 × 300 s) with a voxel size of 0.9 × 0.9 × 0.9 mm, and a 2 mm Gaussian filter.

### Image data processing

**Figure 1:**
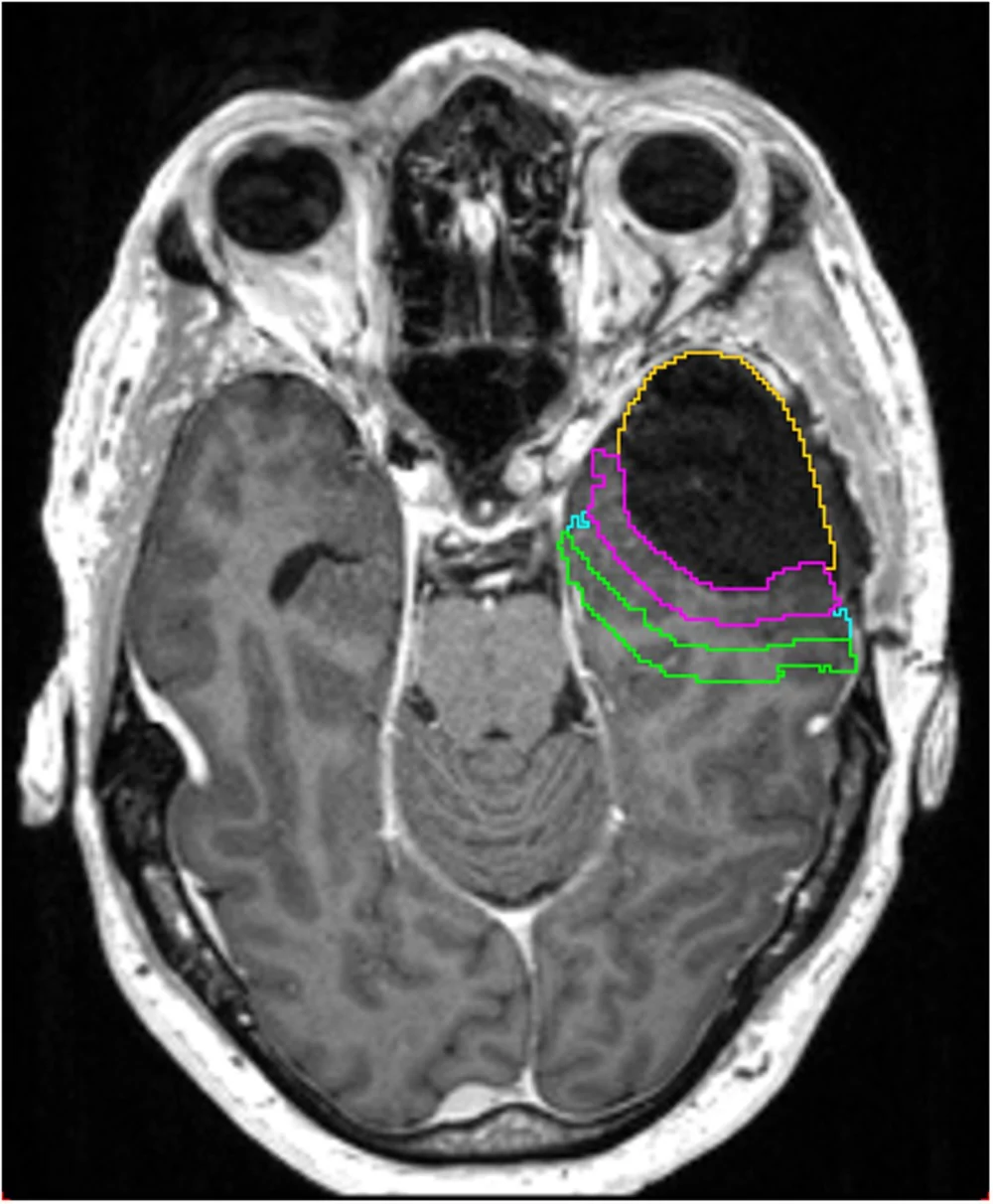
Dilatation of the surgical cavity with the peri-surgical cavity. In orange is the surgical cavity, in pink the peri-surgical cavity 0-5 mm, in light blue the peri-surgical cavity of 5-10 mm, and in green the peri-surgical cavity 10-15 mm.

DWI, IVIM, perfusion, and MET PET data resulted in the generation of their corresponding parameters (see Supplementary Materials). For each parameter, data were extracted for the surgical cavity, peri-surgical cavity 0-5 mm, peri-surgical cavity 5-10 mm, and peri-surgical cavity 10-15 mm (see Figure 1). A cross-wise NAWM sphere with a 10 mm radius was used for control purposes.

### Statistical analysis

First normality was assessed using the Shapiro Wilk test for which a *P < .*05 indicated a non-normal distribution. Differences between regions were determined using either parametric (ANOVA) or non-parametric tests (Kruskal–Wallis test), depending on normality. The parameters were reported with mean and SD, irrespective of normality, to enhance data interpretation, as it is difficult to interpret the data if peri-surgical areas from various distances are not reported with the same variable (ie, median vs mean). Then the various parameters extracted from DWI, IVIM, PWI, and PET were correlated with PFS and OS using Spearman. In addition, the variables were determined on collinearity using Tolerance < 0.1 and variance inflation factor (VIF) >5 for collinearity thresholds. Non-collinear variables were then used to perform Cox regression analysis with OS. If variables were collinear, the variable with the highest correlation coefficient with OS was used for the Cox regression. A *P* value of .05 2-sided was used for all analyses. Due to the explorative nature of this study, we did not correct for multiple comparisons.

## Results

### Demographics

Twenty-one subjects were included in the study (Table 1). Of these 21 subjects, the OS was available for 17 subjects and the PFS for 13 subjects. This is because after treatment in our center, patients were often followed up in peripheral hospitals that were not actively involved in the research and potential involvement was beyond METc approval. All 21 subjects underwent DWI, in 9 of these 21 subjects the DWI was suitable for IVIM, 17 of the 21 underwent DSC MRI, and 13 of the 21 underwent [^11^C]Methionine PET.

**Table 1:** Subject demographics.

Age (year), mean ± SD	61.2 ± 9.0	
Gender (% male)	16M/5F (76.2)	
Progression-free survival time (min) mean ± SD	10.7 ± 9.8	
Overall survival time (min) mean ± SD	13.6 ± 7.1	
Tumor hemisphere (L/R)	7/14	
IDH1 status	Wildtype	21
	Mutated	0
	Unknown	0
IDH2 status	Wildtype	2
	Mutated	0
	Unknown	19
EGFR	Negative	5
	Positive	12
	Unknown	4
PDGFRA	Negative	6
	Positive	11
	Unknown	4
MGMT	Methylated	8
	Unmethylated	9
	Unknown	4

Abbreviations: EGFR = epidermal growth factor receptor; IDH = isocitrate dehydrogenase; MGMT = methylguanine-DNA methyltransferase; PDGFRA = platelet-derived growth factor receptor A.

### DWI parameters

**Figure 2:**
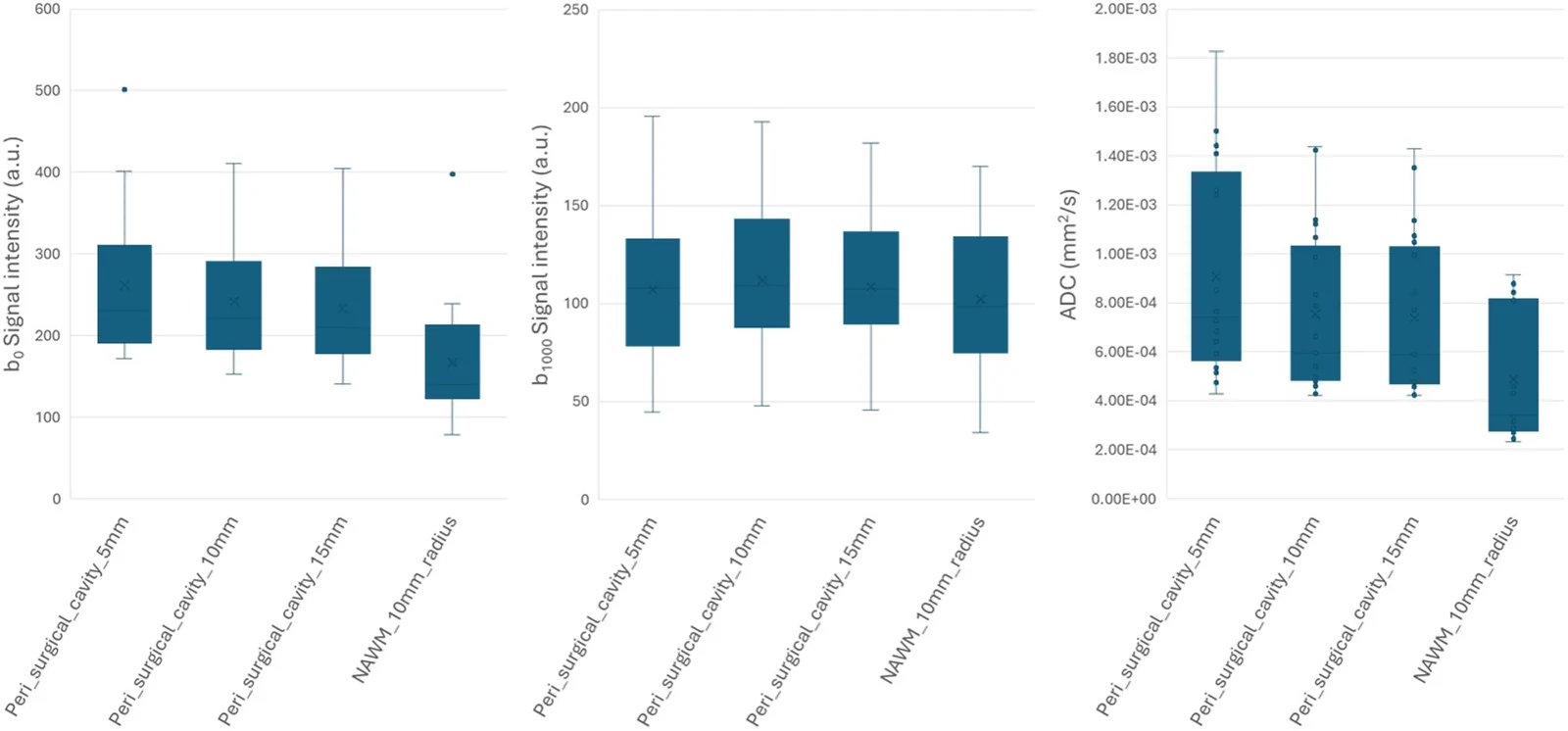
DWI parameters b0, b1000, and ADC for peri-surgical cavity 0-5, 5-10, 10-15 mm, and cross-wise NAWM. Abbreviations: ADC = apparent diffusion coefficient; DWI = diffusion weighted imaging; NAWM = normal appearing white matter.

A gradient from high to low diffusion was observed from peri-surgical cavity 0-5, 5-10, 10-15 mm, to cross-wise NAWM for b0, b1000, and ADC (Figure 2, Table 2, Tables S1-S3). Suggesting that further away from the surgical cavity, the cellular density is higher, which might reflect edema at the surgical cavity, causing a relatively lower cellular density.

**Table 2:** Mean and SD of the assessed imaging parameters per ROI.

	Peri-surgical cavity 0-5 mm	Peri-surgical cavity 5-10 mm	Peri-surgical cavity 10-15 mm	Cross-wise NAWM	*F*	*H*	*P*
b0	262.0 ± 87.2	241.4 ± 77.2	234.0 ± 75.8	166.9 ± 72.6		17.1	<.001
b1000	104.5 ± 42.4	112.0 ± 39.7	108.3 ± 36.4	102.2 ± 38.8	0.23		.874
ADC	0.87 ± 0.43	0.73 ± 0.34	0.71 ± 0.34	0.49 ± 0.26		14.9	.002
D	0.81 ± 0.16	0.75 ± 0.14	0.71 ± 0.11	0.50 ± 0.04		21.8	<.001
D*	21.95 ± 2.18	19.8 ± 1.20	20.8 ± 1.79	17.01 ± 2.37		15.5	.001
*f*	8.21 ± 7.14	7.58 ± 4.75	9.61 ± 5.29	−0.07 ± 0.36		20.3	<.001
MTT	4.54 ± 1.31	4.05 ± 1.27	3.84 ± 1.25	3.68 ± 1.27	1.47		.232
rCBF	43.0 ± 13.8	52.4 ± 12.8	55.5 ± 11.1	26.1 ± 12.1	19.0		<.001
rCBV	2.29 ± 0.63	2.47 ± 0.70	2.53 ± 0.73	1.47 ± 0.57	9.4		<.001
CTH	4.91 ± 1.18	4.61 ± 1.20	4.46 ± 1.21	4.51 ± 1.47		1.7	.642
OEF	0.45 ± 0.08	0.45 ± 0.08	0.44 ± 0.09	0.44 ± 0.12		0.7	.882
CMRO_2_	16.2 ± 4.04	18.5 ± 4.4	19.3 ± 4.4	11.1 ± 4.37	12.5		<.001
SUVmean	1.56 ± 0.87	1.70 ± 0.86	1.60 ± 0.82	1.22 ± 0.84		6.5	.091
SUVmax	4.78 ± 1.73	4.51 ± 1.39	4.07 ± 1.48	2.55 ± 1.75		10.8	<.001

Abbreviations: ADC = apparent diffusion coefficient; CMRO_2_ = cerebral metabolic rate of oxygen; CTH = capillary transit time heterogeneity; D = diffusion coefficient; D* = pseudo-diffusion coefficient; *f =* perfusion fraction; MTT = mean transit time; NAWM = normal appearing white matter; OEF = oxygen extraction fraction; rCBF = relative cerebral blood flow; rCBV = relative cerebral blood volume; SUV = standardized uptake value.

No DWI parameters derived from either cross-wise NAWM, surgical cavity, peri-surgical cavity 0-5, 5-10, or 10-15 mm correlated significantly with OS or PFS (Table S4). Hence, there was also no variable associated with survival time according to Cox regression (Table S5).

### IVIM parameters

**Figure 3:**
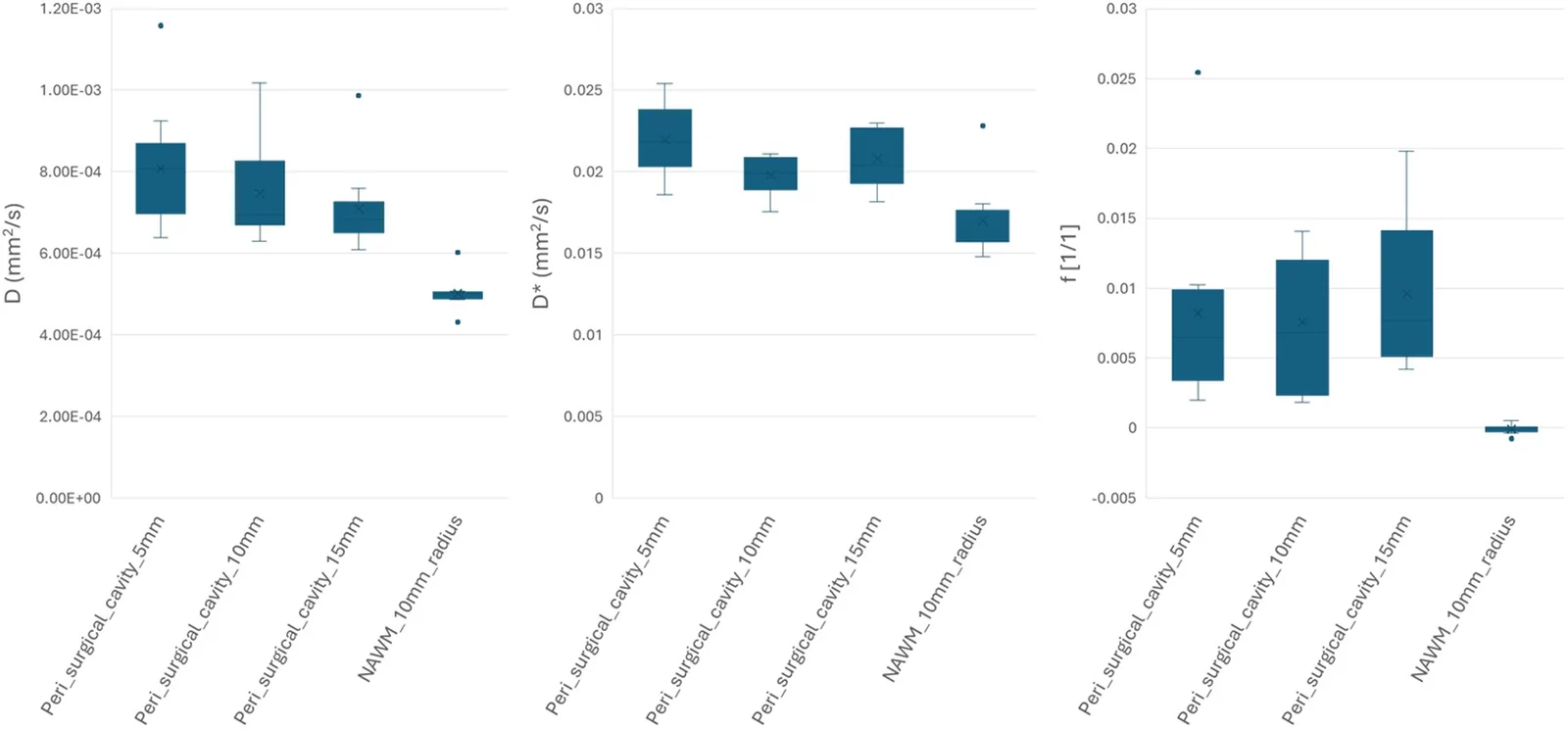
IVIM parameters D, D*, and *f* for peri-surgical cavity 0-5, 5-10, 10-15 mm, and cross-wise NAWM. Abbreviations: D = diffusion coefficient; D* = pseudo-diffusion coefficient; *f* = perfusion fraction; IVIM = intra-voxel incoherent motion; NAWM = normal appearing white matter.

A gradient from high to low diffusion was observed from peri-surgical cavity 0-5, 5-10, 10-15 mm, to cross-wise NAWM for D, but not for D* and *f* (Figure 3, Table 2, Tables S6-S8). Suggesting the further away from the surgical cavity, the higher the cellular density, which most likely reflects edema around the surgical cavity, similar to the DWI findings.

Of the IVIM parameters, D* of peri-surgical cavity 5-10 mm (*r* = −0.786, *P = .*036, *N* = 7) and D of peri-surgical cavity 0-5 mm (*r = *0.821, *P = .*023, *N* = 7) were significantly correlated with OS time. This thus suggests that a lower perfusion (low D*) and a lower cellular density (high D) are associated with a longer survival. In contrast, a higher perfusion (high D*) and a higher cellular density (low D) were associated with a shorter survival. No other IVIM parameters derived from either cross-wise NAWM, surgical cavity, peri-surgical cavity 0-5, 5-10, or 10-15 mm correlated significantly with OS time or progression-free survival time (Table S9). No variable was associated with survival time according to Cox analysis (Table S5).

### DSC parameters

**Figure 4:**
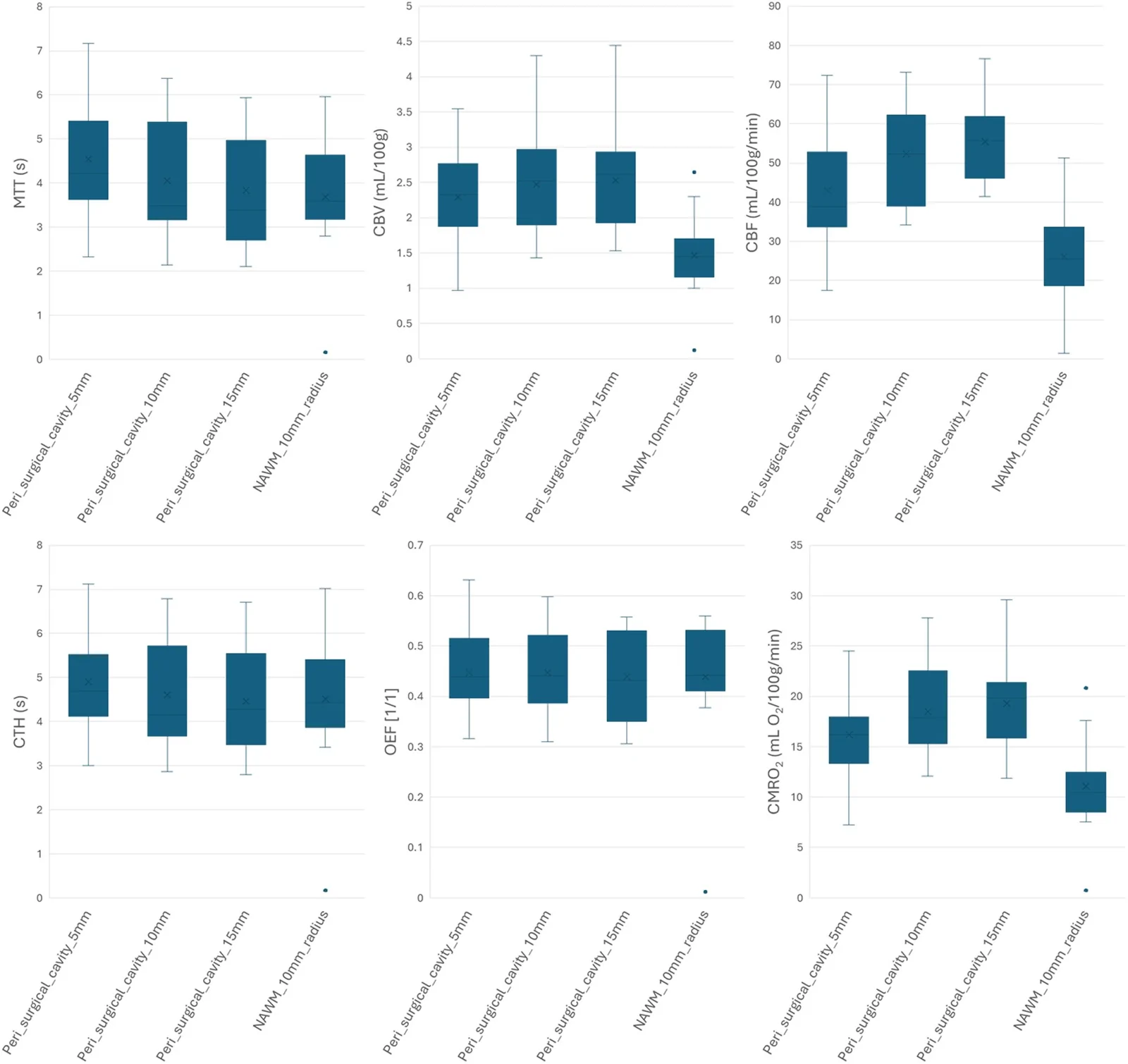
DSC parameters MTT, rCBV, rCBF, CTH, O EF, and CMRO_2_ for peri-surgical cavity 0-5, 5-10, 10-15 mm, and cross-wise NAWM. Abbreviations: CMRO_2_ = cerebral metabolic rate of oxygen; CTH = capillary transit time heterogeneity; DSC = dynamic susceptibility contrast; MTT = mean transit time; NAWM = normal appearing white matter; OEF = oxygen extraction fraction; rCBF = relative cerebral blood flow; rCBV = relative cerebral blood volume.

A gradient from high to low perfusion was observed from peri-surgical cavity 0-5, 5-10, 10-15 mm, to cross-wise NAWM for MTT and CTH, but not for rCBV, rCBF, OEF, and CMRO_2_ (Figure 4, Table 2, Tables S10-S12). This means a prolonged blood transit through the microvasculature in a high heterogeneous manner close to the surgical cavity, in contrast to a short transit time, thus fast blood flow, and a more homogeneous vascular flow further away from the surgical cavity. This could also correspond with distorted blood flow due to surgery.

From the DSC parameters, none of the parameters derived from either cross-wise NAWM, peri-surgical cavity 0-5, 5-10, or 10-15 mm correlated significantly with OS time or progression-free survival time (Table S13). Hence, there was also no variable associated with survival time according to Cox regression (Table S5).

### [^11^C]Methionine PET

**Figure 5:**
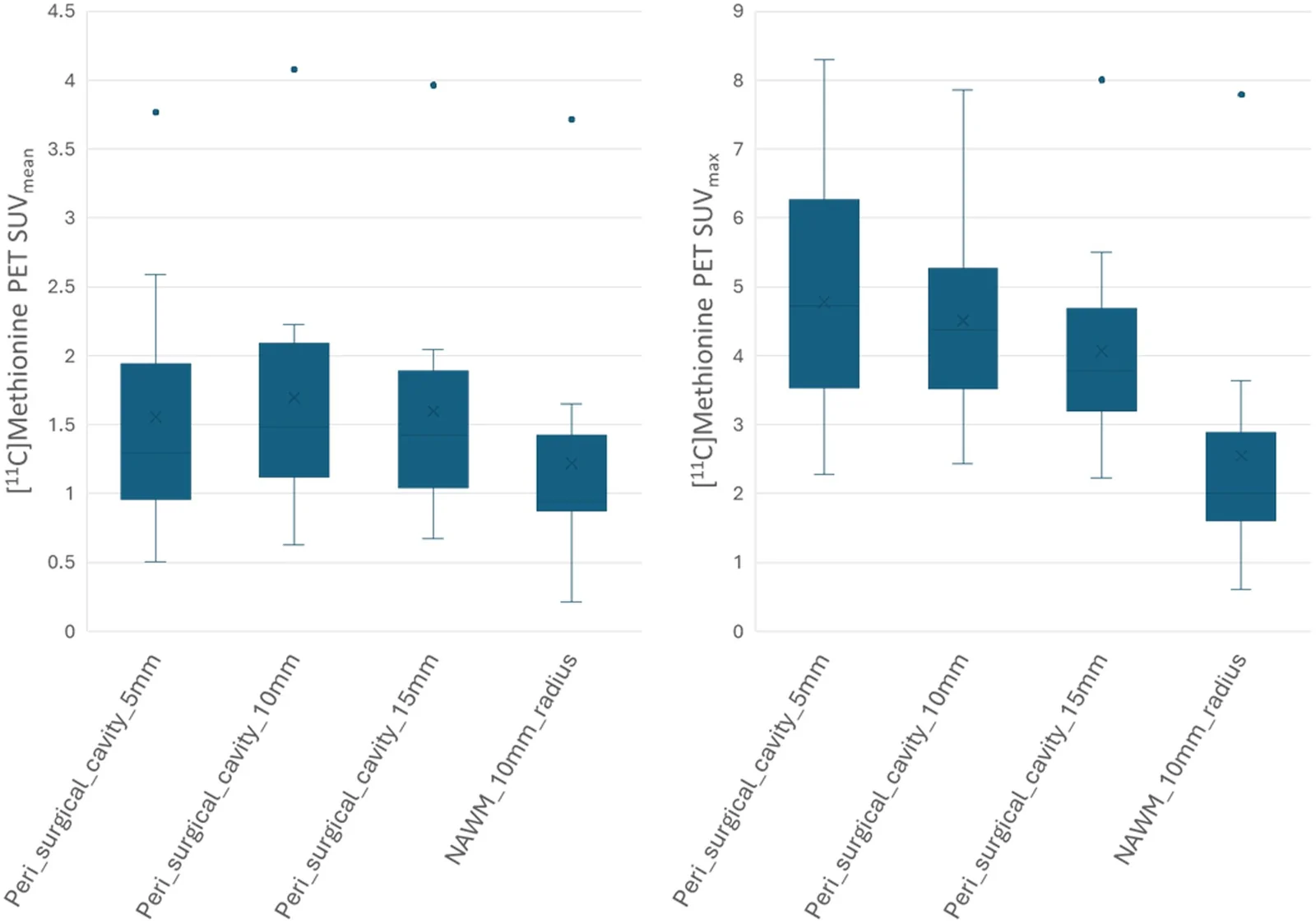
[^11^C]Methionine PET expressed as SUVmean and SUVmax for peri-surgical cavity 0-5, 5-10, 10-15 mm, and cross-wise NAWM. Abbreviations: PET = positron emission tomography; SUV = standardized uptake value.

A gradient from high to low [^11^C]Methionine PET uptake was observed from peri-surgical cavity 0-5, 5-10, 10-15 mm, to cross-wise NAWM for SUVmax, but not for SUVmean (Figure 5, Table 2, Tables S14 and S15). This would mean that there is more metabolic activity around the surgical cavity than further away from the surgical cavity.

[^11^C]Methionine SUVmean uptake and [^11^C]Methionine SUVmax in any of the regions was not significantly correlated with either OS time or progression-free survival (Table S16). In the Cox regression, SUVmax in the 0-5 mm peri-surgical cavity was found to be significantly associated (hazard ratio [HR] = 13.022, *P = .*044) with OS (Table S5). This means that each 1-unit increase in SUVmax in the 0-5 mm peri-surgical cavity was associated with a 13.02-fold increase in the hazard of death. This suggests that patients with higher SUVmax values had a markedly shorter survival time compared to those with lower values.

## Discussion

Prediction of survival time as early as possible, already after surgical removal of the tumor and before subsequent radio- and chemotherapy remains an area of high interest especially due to potential personalized future treatment. Therefore, the aim of this study was to identify an imaging parameter on post-surgical scans that could predict the progression-free survival time or the OS time. Our study shows for OS a strong negative correlation with D* and strong positive correlation with D, probably representing tumor infiltration. These findings illustrate that IVIM could provide valuable insight in OS time based on post-operative scans of patients with GBM.

In general, we expected a gradient of strong correlations with OS with the peri-surgical cavity from 0 to 5 mm to moderate correlations for peri-surgical cavity from 5-10 mm to weak correlations for peri-surgical cavity from 10 to 15 mm, because tumor infiltrations are more common close to the tumor resection location.^14–16^ However, such a gradient was not observed. Only peri-surgical cavity of 0-5 mm was correlated to OS time for D and peri-surgical cavity of 5-10 mm for D*. This suggests that a lower perfusion (low D*) and a lower cellular density (high D) are associated with a longer survival, in contrast, a higher perfusion (high D*) and a higher cellular density (low D) are associated with a shorter survival. This might indicate that both higher cellularity and tissue vascularity could be important predictors for tumor recurrence.

These findings derived from IVIM are also in line with prior diffusion literature showing that lower diffusion metrics in high-grade glioma are generally associated with higher cellularity and poorer outcome.^17–19^ The negative association between D* and OS suggests that higher microvascular perfusion is linked to shorter survival, consistent with the adverse prognostic role of angiogenesis and with prior IVIM work in newly diagnosed glioblastoma showing shorter survival in patients with higher perfusion-related IVIM parameters, including D*.^20^ The fact that D* was significant in the 5-10 mm shell but not in the 0-5 mm shell may indicate that vascular measurements immediately adjacent to the cavity are more strongly confounded by ultra-early post-surgical changes, such as rim restriction/ischemia, vascular injury, blood products, and evolving edema, which are commonly observed on immediate post-operative imaging and may obscure tumor-related perfusion differences in the innermost rim.^21–23^ Taken together, these findings suggest that both residual infiltrative tumor burden and peri-cavity vascularity contribute to prognosis, but that their relative detectability may differ by distance from the cavity in the ultra-early post-operative setting.

Somewhat surprisingly is the poor performance of DSC-derived parameters for predicting OS time. In contrast, the perfusion sensitive DWI method, IVIM, performed pretty well. One of the advantages of IVIM above DSC is the generation of “free” perfusion images with IVIM, as they can be generated from the diffusion images, without the need for additional scanning time. Another advantage is that IVIM does not require the administration of contrast agents to assess perfusion. This is especially beneficial for patients with contraindications like nephrogenic system fibrosis (NSF) or renal failure. In addition, gadolinium deposits have been observed in the brain and other organs of patients having received multiple injections of contrast agents. This leads to a general trend for reducing the number of scans using contrast agents world-wide. The IVIM method, is thus particularly appealing for patients requiring multiple MRI scans, as often is the case in oncology. The high performance for predicting OS time for IVIM as compared to DSC further illustrates the importance of IVIM for routine clinical applications.

Other studies combined conventional MRI scans (T_1_w, T_2_w, T_2_w-FLAIR, T_1_w-post GD) and advanced MRI scans (DSC, DTI) for the prediction of OS time of 101 pre-surgical GBM patients.^24^ With regards to DSC, they calculated rCBV, peak height, and percentage signal recovery. For DTI, they calculated axial diffusivity (AD), radial diffusivity (RD), ADC, and fractional anisotropy (FA). This study found a good prediction when all conventional MRI scans were combined and when all advanced MRI scans were combined. However, they did not combine both conventional and advanced MRI scans.^24^ In contrast, with our study, we performed the analysis with individual parameters and found already a good prediction with some of these parameters, like D, D*, and [^11^C]Methionine uptake. Another study assessing 23 post-surgery GBM patients assessed the prediction of OS time using DSC and dynamic contrast enhanced (DCE) MRI.^25^ For DSC, the rCBF and the rCBV were generated. DCE-derived parameters were unidirectional contrast agent capillary transfer constant (*K*^trans^) and plasma volume (*v*_p_). This study showed that DCE derived parameters were better in predicting OS time than DSC-derived parameters, which is in agreement with the poor results obtained with DSC-derived parameters in our study. Another study with 29 pre-surgery GBM patients assessed the prediction of OS time using T_2_w, T_2_w-FLAIR, T_1_w-post GD, DSC, and DTI.^26^ For DSC, rCBV and rCBF were calculated and for DTI mean diffusivity (MD) and FA. They found a weak negative correlation between rCBV of the tumor and OS time. A study assessing IVIM and DSC in 15 pre-surgery GBM patients, found that D* and *f* were better in predicting OS time than rCBF and rCBV. This is in agreement with our findings that IVIM-derived parameters performed better than DSC-derived parameters. Another study investigating IVIM and DWI in 38 post-surgery GBM patients found a low ADC at baseline and subsequent stability throughout follow-up is a good predictor for disease progression, but found no association of IVIM-derived parameters with OS time.^27^ A study assessing survival prediction of [^18^F]Fluorethyltyrosine PET in 37 pre-surgery GBM patients, found that tumoral uptake of [^18^F]FET could accurately predict the OS time.^28^ As FET and MET are highly comparable, the strong association of MET between SUVmax in 0-5 mm peri-surgical cavity and survival may reflect the potential of amino-acid PET as a prognostic imaging biomarker in post-operative GBM. However, due to the limited sample size, these results should be interpreted cautiously and confirmed in larger, independent datasets.

Across MET PET studies in newly diagnosed GBM, imaging is most often acquired either shortly before surgery (commonly within days of resection; for example, some GBM cohorts performed MET PET within 3 days pre-operatively) to characterize metabolic tumor extent and support prognostic modeling or surgical strategy.^29^^,^^30^ Post-operatively, MET PET is typically obtained in the first 1-3 weeks after surgery (eg, RT-planning series report acquisition within 2 weeks post-op, and other GBM cohorts report a median ∼12 days, range 3-26 days after surgery), while radiotherapy itself generally starts ∼3-6 weeks post-op (with some protocols allowing up to 7 weeks from surgery to the start of radiochemotherapy).^31–33^ Importantly, this planning period precedes adjuvant therapy, which is typically initiated within ∼4-6 weeks (and generally ≤6 weeks) after surgery, and metabolic signal can evolve substantially across these early timepoints.^34–36^ Studies that included an immediate post-operative baseline (≤3 days) demonstrate measurable metabolic changes by early post-treatment follow-up, underscoring that timing can influence both uptake and its interpretation.^36^ In contrast to most prior work, we deliberately targeted the ultra-early ≤72 h post-operative window and applied a concentric peri-cavity (0-5 mm; 5-10 mm) assessment to probe the recurrence-prone rim before adjuvant-therapy effects and later pseudoprogression confound MRI and PET readouts.

The peri-surgical cavity ROIs have a width of 5 mm. This means that especially the peri-surgical cavity from 0 to 5 mm might suffer from partial volume effects due to large tissue differences (soft tissue vs surgical cavity), which would be represented as underestimation of the signal due to absence of signal within the surgical cavity. This would be more relevant for PET than for MRI due to the lower spatial resolution of PET. When looking at the SUVmean, the peri-surgical cavity from 0 to 5 mm has a lower value compared to the other regions, but higher than cross-wise NAWM (Table 2). In contrary, SUVmax is highest in the peri-surgical cavity from 0 to 5 mm. Hence, SUVmean seems indeed to suffer from partial volume effects, whereas SUVmax seems to be unaffected. As SUVmax captures the maximum SUV uptake across the whole ROI, SUVmax is less affected by inclusion of low-signal voxels, making it more robust to partial volume effects. The imaging parameters investigated in this study, particularly those derived from IVIM and MET PET, demonstrate promising potential for integration into routine clinical practice. Parameters such as the diffusion coefficient (D) and pseudo-diffusion coefficient (D*) may enable earlier stratification of GBM patients by expected OS. Importantly, IVIM offers perfusion-sensitive imaging without the need for contrast agents, which is especially valuable for patients with renal impairment or requiring multiple follow-up scans. MET PET uptake values (SUVmean and SUVmax), particularly the link between SUVmax and survival, support further investigation of these metrics. While DWI- and DSC-derived parameters were less consistently predictive. Overall, these results suggest that selected advanced imaging biomarkers may complement existing radiological assessments and help guide personalized treatment strategies, although validation in larger cohorts is essential.

This study has several limitations, one of it is the high heterogeneity in the dataset with regards to the data acquisition. This led to overall, relatively small sample sizes for the individual methods. Therefore, we only assessed the predictive value of the parameters from individual imaging techniques. Another possibilities is to combine all the parameters extracted from the various imaging techniques, which might lead to a stronger prediction of the OS time. However, our data represent a clinical cohort with real-world data and despite the small sample size, we found strong correlations with IVIM for OS time. Although the surgical cavity was removed from all analyses and regions suspicious for residual enhancing tumor were excluded from the peri-cavity masks, other early post-operative biological processes (such as blood products/hemorrhage, inflammation, BBB disruption, and evolving edema), might affect quantitative PET and MRI measurements in this ultra-early window and may have impacted our results. Aside from PET, we did not explore the effects of maximum/minimum parameters for the other modalities but used average values across the various ROIs. This could have led to a loss of visualization of tumor heterogeneity. DSC, DWI, and IVIM all make use of an EPI sequence. EPI sequences are affected by EPI-related susceptibility distortions, which occur especially near air-tissue interfaces, and might have impacted the results.^37^ We used the clinical parameters SUVmean and SUVmax for the PET analysis. A normalized metric, like tumor-to-brain ratio (TBR) for PET quantification reduces variability in uptake times, reconstruction settings, and inter-patient systemic factors. However, our MET PET acquisition was strictly standardized across participants, including a fixed uptake-time window, identical scanner/reconstruction settings, and consistent patient preparation and injection procedures. Under these controlled conditions, SUVmean/SUVmax provide a robust and widely used semiquantitative measure that enables direct comparison across subjects. In addition, we extracted cross-wise NAWM values for every subject and used this region as an internal reference to verify that background uptake was stable across the cohort, thereby reducing the likelihood that inter-patient systemic variability drove the observed associations. While a normalized ratio can be computed mathematically (shell-to-NAWM), in the immediate post-operative setting, where there is no discrete “tumor VOI” by definition, such ratios mainly represent a rescaling of SUV without necessarily improving biological interpretability. For clarity and comparability with prior MET PET GBM literature, we therefore reported SUV metrics. Furthermore, the chosen *b*-value threshold for IVIM processing might have been too arbitrary chosen. However, for brain imaging, it is recommended to use a higher *b* value threshold compared to other tissue types. This is because brain tissue contains a high proportion of small, tightly packed cells, which results in restricted diffusion and a low signal-to-noise ratio at lower *b* values. In addition, several studies have suggested that a *b* value threshold between 100 and 200 s/mm^2^ is optimal for IVIM analysis in the brain. For example, a study by Federau et al.^38^ found that a *b* value of 200 s/mm^2^ was optimal for IVIM analysis of brain tissue in healthy volunteers, based on the highest signal-to-noise ratio and the most stable fitting parameters. Another study by Zhang et al.^39^ also found that a *b* value of 200 s/mm^2^ was optimal for IVIM analysis in brain tumors. As we did not acquire a *b*-value of 200 s/mm^2^, our chosen threshold of 250 s/mm^2^ seems like the best choice.

## Conclusion

In conclusion, our study underscores the potential of IVIM and MET PET imaging parameters in predicting post-surgery survival for GBM patients. Parameters like D, and D* from IVIM and SUVmax from MET PET showed strong associations with OS, indicating their potential predictive utility. These suggest that a lower perfusion (low D*), a lower cellular density (high D), and low SUVmax MET PET are associated with a longer survival and a higher perfusion (high D*), higher cellular density (low D), and high SUVmax MET PET were associated with a shorter survival. Unexpectedly, our findings challenge anticipated correlations based on the peri-surgical cavity distance. The identification of imaging parameters with strong correlations emphasizes the potential for enhanced prognostic accuracy, promising improved patient care of GBM patients, resulting in more personalized treatment strategies.

## Supplementary Material

tqag117_Supplementary_Data

## Data Availability

Due to privacy regulations the clinical data collected in this study are not deposited in a public registry, but the data can be made available via a request to the corresponding author. Anonymized data can be made available after approval of the participants and when a signed data transfer agreement is in place. Software programs used in the study are publicly (FSL, MRtrix, ANTs, MITK) or commercially (Cercare, PMOD, v4.1) available.
