# Supplementary material for: Impact on survival of glioblastoma patients in relation to the imaging of the peri-surgical area: a multi-parametric diffusion MRI, perfusion MRI, and [11C]MET PET study
Source: Br J Radiol. 2026 May 20;99(1185):1871–80. doi: 10.1093/bjr/tqag117 (PMC13489388; doi:10.1093/bjr/tqag117)
Supplement: Supplementary file 1 [file tqag117_supplementary_data.docx]

**Supplementary Data**

DWI and IVIM data processing

The DWI was subjected to noise reduction, corrected for Gibbs ringing artefacts, motion, eddy current, and bias due to MR field inhomogeneity using MRtrix3 (Florey Institute of Neuroscience and Mental Health, Melbourne, Australia), FSL (FMRIB Software Library, Oxford, United Kingdom), and ANTs (University of Pennsylvania, Philadelphia, USA).^21–23^ Then the conventional diffusion parameters (ADC, b 0 s/mm^2^, b 1000 s/mm^2^) and IVIM parameters were extracted from the data using MITK Diffusion (German Cancer Research Center (DKFZ), Heidelberg, Germany).^24^ Since the estimation of the flowing blood fraction (f) relies on accurate diffusion measurements, noise correction improves signal-to-noise ratio and therefore enhances accuracy of diffusion estimation. Correcting Gibbs ringing artifacts, which arise at sharp tissue boundaries (e.g., CSF and brain tissue) due to the limited number of measured MRI frequencies, enhances image quality and can uncover subtle tissue changes that might otherwise remain undetected.^25^ We used simultaneous correction for Eddy currents and subject motion as rapidly changing gradient and RF fields can induce Eddy currents that cause distortions like stretching and shearing in the images^22^. Moreover, correcting B_1_ field inhomogeneities to compensate for resonance frequency errors helped to ensure consistent signal intensity across DW images, supporting more accurate diffusion and IVIM estimations. The simultaneous fitting of all the IVIM parameters is known to be sensitive to noise and may lead to erroneous or inaccurate parameter estimates.^11^ Within the brain, with b-values higher than 250 s/mm^2^, the IVIM effects have disappeared.^26^ The random water displacement results in an exponential decay of the signal amplitude with the degree of field gradient encoding (b value), for medium b values (250-1000 s/mm^2^). This means that for b values between 250 and 1000 s/mm^2^ the tissue diffusion of free water is the prime contributor to the signal decay. The two different biological phenomena as measured with low (<250 s/mm^2^) and medium (250-1000 s/mm^2^) b values can be described according to a bi-exponential model, which separates the diffusion related signal (represented by the slow decaying component) from the perfusion related signal (represented by the fast decaying component) by fitting the diffusion weighted MRI signal to a bi-exponential decay curve. Therefore, medium b-values (250-1000 s/mm^2^) were used to estimate the diffusion coefficient (D), leading to splitting of the signal in two parts, resulting into a 2-step fitting process. This would subsequently improve the parameter estimates, by decreasing the number of unknown variables in the IVIM equation:

$$S_{b}=S_{0}\times\left( 1-f \right) \times e^{-b\times D}+f\times e^{-b \times\left( D +D^{*} \right)}$$

where S_b_ is the diffusion-weighted signal intensity at a given b value, S_0_ is the signal intensity without diffusion weighting, f is the perfusion fraction, and D* is the pseudo-diffusion coefficient representing the perfusion-related signal attenuation.

PWI data processing

The DSC images were corrected for motion and leakage using Cercare Medical Neurosuite (CMN; Cercare Medical, Aarhus, Denmark), release 2023020102.^8,27,28^ Following, AIF generation,^29^ the MTT, rCBV, rCBF, CTH, OEF, and CMRO_2_ were generated using Cercare as published previously.^8,27,28^

MET PET processing

To obtain SUV MET PET images, the detected tracer concentration within a voxel was divided by the ratio of a person’s net injected dose and bodyweight on scanner. The SUV mean was considered the mean SUV of the ROI, and the SUV max was considered the maximum voxel intensity within a ROI.

ROI definition

The surgical cavity after tumour removal was delineated on T_1_w post gadolinium contrast and visually confirmed on accuracy using T_2_w-FLAIR and T_1_w prior gadolinium contrast using PMOD (PMOD Technologies LLC, Zurich, Switzerland). Subsequently, the ROI of the surgical cavity was dilated with 5 mm, 10 mm, and 15 mm. Residual tumour as depicted as hyperintensities on T_1_w post gadolinium contrast MRI scans was omitted from the dilations. The surgical cavity and the dilatations were then transformed into binary masks.

Tissue segmentation was performed using SPM12 (Wellcome Trust Centre for Neuroimaging, London, UK) on T_1_w MRI.^30^ The grey matter (GM) and white matter (WM) were transformed into binary masks using a tissue probability of >0.5. The GM and WM binary masks were combined to create 1 binary mask containing both GM and WM. This was to ensure that the peri-surgical area contained only GM or WM tissue.

In order to create peri-surgical cavity ROIs of a width from 0 to 5 mm, from the binary mask with 5 mm dilatation of the surgical cavity was the binary mask of the surgical cavity subtracted and multiplied by the binary mask containing both GM and WM. The subtraction of the surgical cavity was performed to ensure that only the peri-surgical cavity voxels were included in the ROI formation, and the multiplication with the mask containing both GM and WM was to ensure that the peri-surgical cavity voxels were not confounded by either CSF or other non-brain tissues (Figure 1).

The above procedure for peri-surgical cavity ROIs of a width of 5 mm was repeated for the dilatation of 10 and 15 mm, but then instead of the surgical cavity, the binary mask of either 5 mm dilatation or 10 mm dilatation was used, respectively. This resulted into ROIs covering 0-5 mm of peri-surgical cavity (5 mm dilatation), 5-10 mm of peri-surgical cavity (10 mm dilatation), and 10-15 mm of peri-surgical cavity (15 mm dilatation) (Figure 1).

In short, avoidance of inclusion of non-brain tissue in the construction of the peri-surgical cavity ROIs is as follows. Grey matter (GM) and white matter (WM) were segmented and used as an explicit inclusion mask, and the peri-surgical cavity ROIs were generated as concentric dilations around the cavity ensuring that only voxels belonging to the GM or WM were assigned to the peri-surgical cavity ROIs. Accuracy of GM and WM segmentations, dilatations, and generation of the eventual peri-surgical cavity ROIs were confirmed by visual inspection.

Furthermore, for control purposes, a cross-wise sphere with a 10 mm radius was corrected with ≥0.9 probability for white matter as defined by SPM12 tissue segmentation to generate a cross-wise normal appearing white matter (NAWM) for control purposes.

The processed DWI, IVIM, PWI, and PET images, were aligned to T_1_w post contrast MRI using rigid co-registration with SPM12. Registration accuracy was confirmed visually by an experienced neuro-imaging scientist and neuro-radiologist. Subsequently, the aforementioned ROIs for surgical cavity, peri-surgical cavity 0-5 mm, peri-surgical cavity 5-10 mm, peri-surgical cavity 10-15 mm, and NAWM were loaded onto the co-registered images to extract the mean ROI values.

Supplementary Table 1: Post-Hoc tests of DWI-derived b0

| Sample 1-Sample 2 | Test Statistic | Std. Test Statistic | Sig. |
| --- | --- | --- | --- |
| NAWM vs. 0-5mm peri-surgical cavity | 28.95 | 3.85 | 0.001 |
| NAWM vs. 5-10mm peri-surgical cavity | 23.95 | 3.18 | 0.009 |
| NAWM vs. 10-15mm peri-surgical cavity | 20.52 | 2.73 | 0.038 |
| 10-15mm peri-surgical cavity vs. 0-5mm peri-surgical cavity | 8.43 | 1.12 | 1.000 |
| 5-10mm peri-surgical cavity vs. 0-5mm peri-surgical cavity | 5.00 | 0.66 | 1.000 |
| 10-15mm peri-surgical cavity vs. 5-10mm peri-surgical cavity | 3.43 | 0.46 | 1.000 |

Supplementary Table 2: Post-Hoc tests of DWI-derived b1000

| (I) ROI | (J) ROI | Mean Difference (I-J) | Std. Error | Sig. |
| --- | --- | --- | --- | --- |
| 0-5mm peri-surgical cavity | 5-10mm peri-surgical cavity | -4.97 | 11.95 | 0.679 |
|  | 10-15mm peri-surgical cavity | -1.49 | 11.95 | 0.901 |
|  | NAWM | 4.85 | 11.95 | 0.686 |
| 5-10mm peri-surgical cavity | 0-5mm peri-surgical cavity | 4.97 | 11.95 | 0.679 |
|  | 10-15mm peri-surgical cavity | 3.48 | 11.95 | 0.772 |
|  | NAWM | 9.81 | 11.95 | 0.414 |
| 10-15mm peri-surgical cavity | 0-5mm peri-surgical cavity | 1.49 | 11.95 | 0.901 |
|  | 5-10mm peri-surgical cavity | -3.48 | 11.95 | 0.772 |
|  | NAWM | 6.34 | 11.95 | 0.597 |
| NAWM | 0-5mm peri-surgical cavity | -4.85 | 11.95 | 0.686 |
|  | 5-10mm peri-surgical cavity | -9.81 | 11.95 | 0.414 |
|  | 10-15mm peri-surgical cavity | -6.34 | 11.95 | 0.597 |

Supplementary Table 3: Post-Hoc tests of DWI-derived ADC

| Sample 1-Sample 2 | Test Statistic | Std. Test Statistic | Sig. |
| --- | --- | --- | --- |
| NAWM vs. 0-5mm peri-surgical cavity | 27.91 | 3.71 | 0.001 |
| NAWM vs. 5-10mm peri-surgical cavity | 20.71 | 2.75 | 0.036 |
| NAWM vs. 10-15mm peri-surgical cavity | 18.14 | 2.41 | 0.096 |
| 5-10mm peri-surgical cavity vs. 0-5mm peri-surgical cavity | 7.19 | 0.96 | 1.000 |
| 10-15mm peri-surgical cavity vs. 0-5mm peri-surgical cavity | 9.76 | 1.30 | 1.000 |
| 10-15mm peri-surgical cavity vs. 5-10mm peri-surgical cavity | 2.57 | 0.34 | 1.000 |

Supplementary Table 4: Correlation coefficients of the DWI parameters with progression-free survival and overall survival time

| DWI parameters |  | OS | PFS |
| --- | --- | --- | --- |
| b0_5mm | Correlation | -0.287 | 0.231 |
|  | P | 0.264 | 0.471 |
|  | N | 17 | 12 |
| b1000_5mm | Correlation | -0.159 | 0.021 |
|  | P | 0.541 | 0.948 |
|  | N | 17 | 12 |
| ADC_5mm | Correlation | -0.054 | 0.322 |
|  | P | 0.837 | 0.308 |
|  | N | 17 | 12 |
| b0_10mm | Correlation | -0.27 | 0.42 |
|  | P | 0.295 | 0.175 |
|  | N | 17 | 12 |
| b1000_10mm | Correlation | -0.309 | 0.021 |
|  | P | 0.228 | 0.948 |
|  | N | 17 | 12 |
| ADC_ _10mm | Correlation | -0.088 | 0.028 |
|  | P | 0.736 | 0.931 |
|  | N | 17 | 12 |
| b0_15mm | Correlation | -0.316 | 0.287 |
|  | P | 0.216 | 0.366 |
|  | N | 17 | 12 |
| b1000_15mm | Correlation | -0.326 | -0.014 |
|  | P | 0.202 | 0.966 |
|  | N | 17 | 12 |
| ADC_ _15mm | Correlation | -0.12 | 0.14 |
|  | P | 0.646 | 0.665 |
|  | N | 17 | 12 |
| b0_NAWM_10mm_radius | Correlation | -0.375 | -0.189 |
|  | P | 0.138 | 0.557 |
|  | N | 17 | 12 |
| b1000_NAWM_10mm_radius | Correlation | -0.275 | -0.364 |
|  | P | 0.286 | 0.245 |
|  | N | 17 | 12 |
| ADC_NAWM_10mm_radius | Correlation | -0.275 | 0.133 |
|  | P | 0.286 | 0.681 |
|  | N | 17 | 12 |

Supplementary Table 5: Cox regression of the various parameters with overall survival time

| Parameters | | 0-5mm peri-surgical cavity | | 5-10mm peri-surgical cavity | | 10-15mm peri-surgical cavity | | NAWM | |
| --- | --- | --- | --- | --- | --- | --- | --- | --- | --- |
|  |  | HR | p | HR | p | HR | p | HR | p |
| DWI | b0 | 1.005 | 0.382 | 1.005 | 0.596 | 1.004 | 0.685 | 1.007 | 0.059 |
|  | b1000 | 0.999 | 0.922 | 1.004 | 0.872 | 1.004 | 0.885 | n.a. | n.a. |
|  | ADC | 0.000 | 0.788 | 1.789E+131 | 0.892 | 8.6E+163 | 0.875 | n.a. | n.a. |
| IVIM | D | 0.000 | 0.165 | - | 0.297 | - | 0.436 | 0 | 0.762 |
|  | D* | 0.000 | 0.894 | - | 0.178 | - | 0.129 | 7.112E+151 | 0.380 |
|  | f | 1.055E+35 | 0.336 | 0.000 | 0.580 | 72.842 | 0.970 | - | 0.852 |
| DSC | MTT | n.a. | n.a. | n.a. | n.a. | n.a. | n.a. | n.a. | n.a. |
|  | rCBF | n.a. | n.a. | 1.045 | 0.249 | n.a. | n.a. | 1.014 | 0.610 |
|  | rCBV | n.a. | n.a. | n.a. | n.a. | 2.010 | 0.296 | n.a. | n.a. |
|  | CTH | n.a. | n.a. | n.a. | n.a. | n.a. | n.a. | n.a. | n.a. |
|  | OEF | 0.117 | 0.648 | n.a. | n.a. | n.a. | n.a. | n.a. | n.a. |
|  | CMRO2 | n.a. | n.a. | n.a. | n.a. | n.a. | n.a. | n.a. | n.a. |
| PET | SUVmean | 0.002 | 0.060 | 0.039 | 0.148 | 0.143 | 0.238 | 0.143 | 0.238 |
|  | SUVmax | 13.022 | 0.044 | 4.375 | 0.383 | 0.810 | 0.853 | 0.810 | 0.853 |

Supplementary Table 6: Post-Hoc tests of IVIM-derived D

| Sample 1-Sample 2 | Test Statistic | Std. Test Statistic | Sig. |
| --- | --- | --- | --- |
| NAWM vs. 0-5mm peri-surgical cavity | 21.67 | 4.36 | 0.000 |
| NAWM vs. 5-10mm peri-surgical cavity | 18.00 | 3.62 | 0.002 |
| NAWM vs. 10-15mm peri-surgical cavity | 14.33 | 2.89 | 0.023 |
| 5-10mm peri-surgical cavity vs. 0-5mm peri-surgical cavity | 3.67 | 0.74 | 1.000 |
| 10-15mm peri-surgical cavity vs. 0-5mm peri-surgical cavity | 7.33 | 1.48 | 0.839 |
| 10-15mm peri-surgical cavity vs. 5-10mm peri-surgical cavity | 3.67 | 0.74 | 1.000 |

Supplementary Table 7: Post-Hoc tests of IVIM-derived D*

| Sample 1-Sample 2 | Test Statistic | Std. Test Statistic | Sig. |
| --- | --- | --- | --- |
| NAWM vs. 0-5mm peri-surgical cavity | 18.67 | 3.76 | 0.001 |
| NAWM vs. 5-10mm peri-surgical cavity | 10.33 | 2.08 | 0.225 |
| NAWM vs. 10-15mm peri-surgical cavity | 14.33 | 2.89 | 0.023 |
| 5-10mm peri-surgical cavity vs. 0-5mm peri-surgical cavity | 8.33 | 1.68 | 0.560 |
| 10-15mm peri-surgical cavity vs. 0-5mm peri-surgical cavity | 4.33 | 0.87 | 1.000 |
| 5-10mm peri-surgical cavity vs. 10-15mm peri-surgical cavity | -4.00 | -0.81 | 1.000 |

Supplementary Table 8: Post-Hoc tests of IVIM-derived f

| Sample 1-Sample 2 | Test Statistic | Std. Test Statistic | Sig. |
| --- | --- | --- | --- |
| NAWM vs. 0-5mm peri-surgical cavity | 16.67 | 3.36 | 0.005 |
| NAWM vs. 5-10mm peri-surgical cavity | 17.22 | 3.47 | 0.003 |
| NAWM vs. 10-15mm peri-surgical cavity | 20.11 | 4.05 | 0.000 |
| 0-5mm peri-surgical cavity vs. 5-10mm peri-surgical cavity | -0.56 | -0.11 | 1.000 |
| 0-5mm peri-surgical cavity vs. 10-15mm peri-surgical cavity | -3.44 | -0.69 | 1.000 |
| 5-10mm peri-surgical cavity vs. 10-15mm peri-surgical cavity | -2.89 | -0.58 | 1.000 |

Supplementary Table 9: Correlation coefficients of the IVIM parameters with progression-free survival and overall survival time

| IVIM parameters |  | OS | PFS |
| --- | --- | --- | --- |
| D_5mm | Correlation | .821* | 1.000** |
|  | P | 0.023 | . |
|  | N | 7 | 4 |
| Dstar_5mm | Correlation | 0.107 | 0.2 |
|  | P | 0.819 | 0.8 |
|  | N | 7 | 4 |
| f_5mm | Correlation | 0.214 | 0.4 |
|  | P | 0.645 | 0.6 |
|  | N | 7 | 4 |
| D_10mm | Correlation | 0.5 | 0.2 |
|  | P | 0.253 | 0.8 |
|  | N | 7 | 4 |
| Dstar_10mm | Correlation | -.786* | -0.8 |
|  | P | 0.036 | 0.2 |
|  | N | 7 | 4 |
| f_10mm | Correlation | -0.429 | -1.000** |
|  | P | 0.337 | . |
|  | N | 7 | 4 |
| D_15mm | Correlation | 0.429 | 0.8 |
|  | P | 0.337 | 0.2 |
|  | N | 7 | 4 |
| Dstar_15mm | Correlation | -0.571 | -0.8 |
|  | P | 0.18 | 0.2 |
|  | N | 7 | 4 |
| f_15mm | Correlation | -0.214 | -0.8 |
|  | P | 0.645 | 0.2 |
|  | N | 7 | 4 |
| D_NAWM_10mm_radius | Correlation | 0.25 | -0.2 |
|  | P | 0.589 | 0.8 |
|  | N | 7 | 4 |
| Dstar_NAWM_10mm_radius | Correlation | -0.5 | -0.8 |
|  | P | 0.253 | 0.2 |
|  | N | 7 | 4 |
| f_NAWM_10mm_radius | Correlation | 0.214 | -0.4 |
|  | P | 0.645 | 0.6 |
|  | N | 7 | 4 |

Supplementary Table 10: Post-Hoc tests of DSC-derived MTT, rCBF, rCBV, and CMRO_2_

| Variable | (I) ROI | (J) ROI | Mean Difference (I-J) | Std. Error | Sig. |
| --- | --- | --- | --- | --- | --- |
| MTT | 0-5mm peri-surgical cavity | 5-10mm peri-surgical cavity | 0.49 | 0.44 | 0.270 |
|  |  | 10-15mm peri-surgical cavity | 0.71 | 0.44 | 0.112 |
|  |  | NAWM | 0.86 | 0.44 | 0.054 |
|  | 5-10mm peri-surgical cavity | 0-5mm peri-surgical cavity | -0.49 | 0.44 | 0.270 |
|  |  | 10-15mm peri-surgical cavity | 0.22 | 0.44 | 0.619 |
|  |  | NAWM | 0.37 | 0.44 | 0.399 |
|  | 10-15mm peri-surgical cavity | 0-5mm peri-surgical cavity | -0.71 | 0.44 | 0.112 |
|  |  | 5-10mm peri-surgical cavity | -0.22 | 0.44 | 0.619 |
|  |  | NAWM | 0.15 | 0.44 | 0.728 |
|  | NAWM | 0-5mm peri-surgical cavity | -0.86 | 0.44 | 0.054 |
|  |  | 5-10mm peri-surgical cavity | -0.37 | 0.44 | 0.399 |
|  |  | 10-15mm peri-surgical cavity | -0.15 | 0.44 | 0.728 |
| rCBF | 0-5mm peri-surgical cavity | 5-10mm peri-surgical cavity | -9.40 | 4.28 | 0.032 |
|  |  | 10-15mm peri-surgical cavity | -12.50 | 4.28 | 0.005 |
|  |  | NAWM | 16.86 | 4.28 | <0.001 |
|  | 5-10mm peri-surgical cavity | 0-5mm peri-surgical cavity | 9.40 | 4.28 | 0.032 |
|  |  | 10-15mm peri-surgical cavity | -3.10 | 4.28 | 0.471 |
|  |  | NAWM | 26.26 | 4.28 | <0.001 |
|  | 10-15mm peri-surgical cavity | 0-5mm peri-surgical cavity | 12.50 | 4.28 | 0.005 |
|  |  | 5-10mm peri-surgical cavity | 3.10 | 4.28 | 0.471 |
|  |  | NAWM | 29.36 | 4.28 | <0.001 |
|  | NAWM | 0-5mm peri-surgical cavity | -16.86 | 4.28 | <0.001 |
|  |  | 5-10mm peri-surgical cavity | -26.26 | 4.28 | <0.001 |
|  |  | 10-15mm peri-surgical cavity | -29.36 | 4.28 | <0.001 |
| rCBV | 0-5mm peri-surgical cavity | 5-10mm peri-surgical cavity | -0.18 | 0.23 | 0.427 |
|  |  | 10-15mm peri-surgical cavity | -0.24 | 0.23 | 0.301 |
|  |  | NAWM | 0.83 | 0.23 | <0.001 |
|  | 5-10mm peri-surgical cavity | 0-5mm peri-surgical cavity | 0.18 | 0.23 | 0.427 |
|  |  | 10-15mm peri-surgical cavity | -0.06 | 0.23 | 0.809 |
|  |  | NAWM | 1.01 | 0.23 | <0.001 |
|  | 10-15mm peri-surgical cavity | 0-5mm peri-surgical cavity | 0.24 | 0.23 | 0.301 |
|  |  | 5-10mm peri-surgical cavity | 0.06 | 0.23 | 0.809 |
|  |  | NAWM | 1.06 | 0.23 | <0.001 |
|  | NAWM | 0-5mm peri-surgical cavity | -0.83 | 0.23 | <0.001 |
|  |  | 5-10mm peri-surgical cavity | -1.01 | 0.23 | <0.001 |
|  |  | 10-15mm peri-surgical cavity | -1.06 | 0.23 | <0.001 |
| CMRO_2_ | 0-5mm peri-surgical cavity | 5-10mm peri-surgical cavity | -2.29 | 1.47 | 0.125 |
|  |  | 10-15mm peri-surgical cavity | -3.09 | 1.47 | 0.04 |
|  |  | NAWM | 5.11 | 1.47 | <0.001 |
|  | 5-10mm peri-surgical cavity | 0-5mm peri-surgical cavity | 2.29 | 1.47 | 0.125 |
|  |  | 10-15mm peri-surgical cavity | -0.80 | 1.47 | 0.588 |
|  |  | NAWM | 7.40 | 1.47 | <0.001 |
|  | 10-15mm peri-surgical cavity | 0-5mm peri-surgical cavity | 3.09 | 1.47 | 0.04 |
|  |  | 5-10mm peri-surgical cavity | 0.80 | 1.47 | 0.588 |
|  |  | NAWM | 8.20 | 1.47 | <0.001 |
|  | NAWM | 0-5mm peri-surgical cavity | -5.11 | 1.47 | <0.001 |
|  |  | 5-10mm peri-surgical cavity | -7.40 | 1.47 | <0.001 |
|  |  | 10-15mm peri-surgical cavity | -8.20 | 1.47 | <0.001 |

Supplementary Table 11: Post-Hoc test of DSC-derived CTH

| Sample 1-Sample 2 | Test Statistic | Std. Test Statistic | Sig. |
| --- | --- | --- | --- |
| 10-15mm peri-surgical cavity vs. 5-10mm peri-surgical cavity | 2.00 | 0.30 | 1.000 |
| 10-15 mm peri-surgical cavity vs. NAWM | -4.65 | -0.69 | 1.000 |
| 10-15mm peri-surgical cavity vs. 0-5mm peri-surgical cavity | 8.29 | 1.22 | 1.000 |
| 5-10mm peri-surgical cavity vs. NAWM | -2.65 | -0.39 | 1.000 |
| 5-10mm peri-surgical cavity vs. 0-5mm peri-surgical cavity | 6.29 | 0.93 | 1.000 |
| NAWM vs. 0-5mm peri-surgical cavity | 3.65 | 0.54 | 1.000 |

Supplementary Table 12: Post-Hoc test of DSC-derived OEF

| Sample 1-Sample 2 | Test Statistic | Std. Test Statistic | Adj. Sig.a |
| --- | --- | --- | --- |
| 10-15mm peri-surgical cavity vs. 5-10mm peri-surgical cavity | 1.12 | 0.17 | 1.000 |
| 10-15mm peri-surgical cavity vs. 0-5mm peri-surgical cavity | 1.35 | 0.20 | 1.000 |
| 10-15 mm peri-surgical cavity vs. NAWM | -5.18 | -0.76 | 1.000 |
| 5-10mm peri-surgical cavity vs. 0-5mm peri-surgical cavity | 0.24 | 0.04 | 1.000 |
| 5-10mm peri-surgical cavity vs. NAWM | -4.06 | -0.60 | 1.000 |
| 0-5mm peri-surgical cavity vs. NAWM | -3.82 | -0.56 | 1.000 |

Supplementary Table 13: Correlation coefficients of the DSC parameters with progression-free survival and overall survival time

| DSC parameters |  | OS | PFS |
| --- | --- | --- | --- |
| CTH_5mm | Correlation | -0.121 | -0.133 |
|  | P | 0.694 | 0.732 |
|  | N | 13 | 9 |
| MTT_5mm | Correlation | -0.165 | -0.15 |
|  | P | 0.59 | 0.7 |
|  | N | 13 | 9 |
| OEF_5mm | Correlation | 0.258 | -0.05 |
|  | P | 0.394 | 0.898 |
|  | N | 13 | 9 |
| rCBF_5mm | Correlation | -0.077 | -0.3 |
|  | P | 0.803 | 0.433 |
|  | N | 13 | 9 |
| rCBV_5mm | Correlation | -0.06 | -0.167 |
|  | P | 0.845 | 0.668 |
|  | N | 13 | 9 |
| CMRO2_5mm | Correlation | -0.071 | -0.133 |
|  | P | 0.817 | 0.732 |
|  | N | 13 | 9 |
| CTH_10mm | Correlation | 0.060 | 0.000 |
|  | P | 0.845 | 1.000 |
|  | N | 13 | 9 |
| MTT_10mm | Correlation | 0.060 | 0.017 |
|  | P | 0.845 | 0.966 |
|  | N | 13 | 9 |
| OEF_10mm | Correlation | 0.126 | -0.050 |
|  | P | 0.681 | 0.898 |
|  | N | 13 | 9 |
| rCBF_10mm | Correlation | -0.176 | -0.167 |
|  | P | 0.566 | 0.668 |
|  | N | 13 | 9 |
| rCBV_10mm | Correlation | -0.258 | -0.100 |
|  | P | 0.394 | 0.798 |
|  | N | 13 | 9 |
| CMRO2_10mm | Correlation | -0.242 | -0.200 |
|  | P | 0.426 | 0. 606 |
|  | N | 13 | 9 |
| CTH_15mm | Correlation | 0.066 | 0.033 |
|  | P | 0.831 | 0.932 |
|  | N | 13 | 9 |
| MTT_15mm | Correlation | 0.027 | 0.05 |
|  | P | 0.929 | 0.898 |
|  | N | 13 | 9 |
| OEF_15mm | Correlation | 0.033 | 0.05 |
|  | P | 0.915 | 0.898 |
|  | N | 13 | 9 |
| rCBF_15mm | Correlation | -0.313 | -0.067 |
|  | P | 0.297 | 0.865 |
|  | N | 13 | 9 |
| rCBV_15mm | Correlation | -0.28 | 0.083 |
|  | P | 0.354 | 0.831 |
|  | N | 13 | 9 |
| CMRO2_15mm | Correlation | -0.258 | -0.233 |
|  | P | 0.394 | 0.546 |
|  | N | 13 | 9 |
| CTH_NAWM_10mm_radius | Correlation | 0.352 | -0.067 |
|  | P | 0.239 | 0.865 |
|  | N | 13 | 9 |
| MTT_NAWM_10mm_radius | Correlation | 0.28 | -0.15 |
|  | P | 0.354 | 0.7 |
|  | N | 13 | 9 |
| OEF_NAWM_10mm_radius | Correlation | 0.264 | -0.15 |
|  | P | 0.384 | 0.7 |
|  | N | 13 | 9 |
| rCBF_NAWM_10mm_radius | Correlation | -0.374 | 0.05 |
|  | P | 0.209 | 0.898 |
|  | N | 13 | 9 |
| rCBV_NAWM_10mm_radius | Correlation | -0.06 | 0.183 |
|  | P | 0.845 | 0.637 |
|  | N | 13 | 9 |
| CMRO2_NAWM_10mm_radius | Correlation | -0.214 | -0.017 |
|  | P | 0.482 | 0.966 |
|  | N | 13 | 9 |

Supplementary Table 14: Post-Hoc test of SUVmean

| [^11^C]Methionine PET uptakeSample 1-Sample 2 | Test Statistic | Std. Test Statistic | Sig. |
| --- | --- | --- | --- |
| NAWM vs. 0-5mm peri-surgical cavity | 9.4 | 1.6 | 0.686 |
| NAWM vs. 5-10mm peri-surgical cavity | 14.1 | 2.4 | 0.107 |
| NAWM vs. 10-15mm peri-surgical cavity | 11.8 | 2.0 | 0.286 |
| 0-5mm peri-surgical cavity vs. 5-10mm peri-surgical cavity | -4.7 | -0.8 | 1 |
| 0-5mm peri-surgical cavity vs. 10-15mm peri-surgical cavity | -2.4 | -0.4 | 1 |
| 10-15mm peri-surgical cavity vs. 5-10mm peri-surgical cavity | 2.3 | 0.4 | 1 |

Supplementary Table 15: Post-Hoc test of SUVmax [^11^C]Methionine PET uptake

| (I) ROI | (J) ROI | Mean Difference (I-J) | Sig. |
| --- | --- | --- | --- |
| 0-5mm peri-surgical cavity | 5-10mm peri-surgical cavity | 0.09 | 0.646 |
|  | 10-15mm peri-surgical cavity | 0.28 | 0.134 |
|  | NAWM | 0.94 | <0.001 |
| 5-10mm peri-surgical cavity | 0-5mm peri-surgical cavity | -0.09 | 0.646 |
|  | 10-15mm peri-surgical cavity | 0.20 | 0.293 |
|  | NAWM | 0.86 | <0.001 |
| 10-15mm peri-surgical cavity | 0-5mm peri-surgical cavity | -0.28 | 0.134 |
|  | 5-10mm peri-surgical cavity | -0.20 | 0.293 |
|  | NAWM | 0.66 | <0.001 |
| NAWM | 0-5mm peri-surgical cavity | -0.94 | <0.001 |
|  | 5-10mm peri-surgical cavity | -0.86 | <0.001 |
|  | 10-15mm peri-surgical cavity | -0.66 | <0.001 |

Supplementary Table 16: Correlation coefficients of the MET PET parameters with progression-free survival and overall survival time

| [^11^C]MET PET parameters |  | OS | PFS |
| --- | --- | --- | --- |
| PET_mean_5mm | Correlation | 0.309 | 0.095 |
|  | P | 0.355 | 0.823 |
|  | N | 11 | 8 |
| PET_mean_10mm | Correlation | 0.364 | 0.357 |
|  | P | 0.272 | 0.385 |
|  | N | 11 | 8 |
| PET_mean_15mm | Correlation | 0.473 | 0.357 |
|  | P | 0.142 | 0.385 |
|  | N | 11 | 8 |
| PET_mean_NAWM_10mm_radius | Correlation | 0.382 | 0.357 |
|  | P | 0.247 | 0.385 |
|  | N | 11 | 8 |
| PET_max_5mm | Correlation | -0.009 | -0.048 |
|  | P | 0.979 | 0.911 |
|  | N | 11 | 8 |
| PET_max_10mm | Correlation | 0.173 | 0.286 |
|  | P | 0.612 | 0.493 |
|  | N | 11 | 8 |
| PET_max_15mm | Correlation | 0.073 | 0.214 |
|  | P | 0.832 | 0.61 |
|  | N | 11 | 8 |
| PET_max_NAWM_10mm_radius | Correlation | 0.391 | 0.31 |
|  | P | 0.235 | 0.456 |
|  | N | 11 | 8 |

Supplementary Figure 1: Overlay of [^11^C]Methionine PET expressed in SUV on top of a T_1_w MRI post GD injection.

Supplementary Figure 2: Scatter plot between IVIM D for peri-surgical cavity 0-5 mm and overall survival time.

Supplementary Figure 3: Scatter plot between IVIM D* for peri-surgical cavity 5-10 mm and overall survival time.

Supplementary Figure 4: Scatter plot between SUVmax for peri-surgical cavity 0-5 mm and overall survival time.
